# Voices of Loyal Members: Dual Role of Organizational Identification in the Process of Employee Voice

**DOI:** 10.3390/bs15020109

**Published:** 2025-01-22

**Authors:** Yongsuhk Jung, Yongjun Choi

**Affiliations:** 1 Department of Management, Republic of Korea Air Force Academy, Cheongju 28187, Republic of Korea; 2 College of Business Administration, Hongik University, Seoul 04066, Republic of Korea; yongjun.choi@hongik.ac.kr

**Keywords:** employee voice, organizational identification, performance appraisal, theory of loyalty, normative conflict model

## Abstract

Based on Hirschman’s theory of loyalty and Packer’s normative conflict model, the present study examined the roles of organizational identification in the voice emergence and reaction processes, wherein individuals provide voice and receive evaluations for their voice behavior, respectively. Using a survey method, data were collected from 455 cadets and their supervisors at a military educational institute in South Korea, who live and work together under an honor-based organizational system that encourages voice behavior through formal and informal channels. Structural equation modeling (SEM) was used for hypothesis testing. Our findings from multi-source data demonstrated that, when controlling for two social exchange variables (i.e., leader–member exchange and perceived organizational support), organizational identification not only increases voice behavior but also strengthens the positive relationship between voice behavior and supervisor performance evaluations. Specifically, voice behavior has a positive relationship with performance appraisal only when organizational identification is high. Theoretical and practical implications of the findings and directions for future research are discussed.

## 1. Introduction

Organizational members’ voice behavior, defined as discretionary verbal input that is intended to be constructive (E. W. Morrison, 2011; Van Dyne & LePine, 1998), such as providing opinions, suggestions, and ideas, is integral for organizational adaptation and effectiveness (Greenberg & Edwards, 2009; E. W. Morrison, 2011). Past studies on voice behavior have identified a variety of underlying factors, ranging from prosocial motives focused on promoting the organization (E. W. Morrison, 2014) to impression management motives driven by self-serving goals such as gaining recognition or advancing one’s career (Choi et al., 2015; Fuller et al., 2007). These motives are not mutually exclusive; instead, employees often weigh both personal and collective interests when deciding whether to speak up (Grant & Ashford, 2008). This study focuses on prosociality as a main driver for voice behavior because it closely aligns with organizational identification, emphasizing an individual’s integration of organizational goals and values into their sense of self (Riketta, 2005). That is, in contrast with impression management motives, which tend to be self-serving, prosocial motives signify an individual’s genuine commitment to advancing collective interests. This distinction is particularly relevant for understanding how loyalty and identification shape voice behavior. Such an emphasis is also congruent with prior research, suggesting that prosocially motivated voice is more likely to be perceived by managers as both constructive and credible (Van Dyne et al., 1995). Therefore, by focusing on prosocial motives, this study aims to shed light on how identification-driven loyalty can both encourage employees to engage in voice behavior and enhance managerial evaluations of their performance.

One of the notable features of voice behavior is that organizational members must be willing to engage in potential risks to raise their voice. Specifically, because voice behavior challenges the status quo, it may provoke defensive reactions from other organizational members, leading to retaliation, particularly in the form of negative performance evaluations from supervisors (Milliken et al., 2003). Indeed, there is substantial evidence that exercising voice at work can be hazardous (Burris, 2012; Fast et al., 2013) and that employees are acutely aware of these risks (Detert & Edmondson, 2011; Milliken et al., 2003). Even suggestions related to the pursuit of new opportunities for the organization may be met defensively if they conflict with existing plans, require substantial resources, or appear to be risky. This issue is critical for organizations because, if employees remain silent due to the potential negative consequences resulting from exercising voice, they may miss opportunities to enhance organizational effectiveness. In addition, if supervisors fail to receive their subordinates’ voice openly, organizations may face negative consequences, such as increasing work-related injuries (Tucker & Turner, 2015). However, little is known about what could facilitate employee voice behaviors by helping them receive favorable evaluations from their managers.

The present study proposes that individuals’ strong loyalty to their organizations, particularly in the form of organizational identification (Effelsberg & Sulga, 2015; Klehe et al., 2013; van Knippenberg & van Schie, 2000), which refers to the extent to which an “organizational member has linked his or her organizational membership to his or her self-concept, either cognitively (e.g., feeling a part of the organization; internalizing organizational values), emotionally (pride in membership), or both” (Riketta, 2005, p. 361), as a key factor in creating the aforementioned condition. In politics, for example, loyal members tend to offer critiques and propose solutions because of their loyalty, and their loyalty makes their voice more credible. Specifically, in the United States, every five years, politicians from across the political spectrum attempt to prove their patriotic fervor by convincing a sufficient number of people that the country has failed and that their proposed solutions will get the country back on track. As a result, some of these politicians are amply celebrated and ultimately deemed as competent for the job of president. Patriotism, in this context, is an expression of identification with one’s country (Huddy & Khatib, 2007). It communicates to voters that the politician is on their side, even when the politician is criticizing, for instance, voters’ previous choices of candidates.

However, it is not clear if loyalty works the same way in organizational settings. To date, it remains unclear whether the loyalty of organizational members motivates them to participate in voice behavior and, at the same time, to be positively evaluated for their voice, as in the case of loyal politicians. Prior studies on voice behavior have given some attention to whether loyalty results in voice (Burris et al., 2008; Liu et al., 2010), but the findings have been mixed. A recent meta-analysis showed that organizational identification leads to promotive and prohibitive voice behaviors (Chamberlin et al., 2017), which aligns with recent meta-analytic findings suggesting that organizational identification is positively related to an employee’s extra-role performance (Lee et al., 2015). Thus, we aim to address these inconsistencies through the lens of Hirschman’s (1970) theory of exit, voice, and loyalty, which explores the role of loyalty in the decision to exercise voice and its efficacy.

In this study, we argue that the type of loyalty is important and that, in order to establish the significance of social identity, the role of social exchange processes needs to be simultaneously considered. In addition, we propose a dual role for organizational identification. As with politicians, the engagement of strongly identified members in voice is expected to result in payoffs not available to weakly identified organizational members. Thus, grounded in Hirschman’s (1970) theory of loyalty, we expect that people who identify with their organization are more likely to speak up and will be rewarded for doing so. We also draw on Packer’s (2008) model of normative conflict in groups, which describes when and how highly identified members will break from conformity and speak up about collective norms that they believe conflict with the actual or desired identity of the group.

The contributions of this research are twofold. First, it clarifies the relevance of loyalty to voice and provides a deeper understanding of the social processes that influence both engagement in and managerial reactions to voice. In doing so, we answer the recent calls for research on how supervisors respond to their followers’ voice behaviors (Bashshur & Oc, 2015). Second, it further illuminates boundary conditions around the relationship between voice and performance appraisal. The relationships examined here are not only theoretically important but also practically relevant. Whereas most individual difference variables related to voice and voice outcomes, such as personality (LePine & Van Dyne, 2001) and negative affectivity (Grant et al., 2009), tend to be relatively fixed, organizational identification is a psychological state that can be influenced by leaders (Epitropaki & Martin, 2005; Hogg, 2001; Walumbwa et al., 2011). Thus, it may be a primary mechanism through which leadership affects both the quantity and quality of voice. This, in turn, has implications for the organization’s well-being and the employee’s performance and career development.

## 2. Theory and Hypotheses

### 2.1. Normative Conflict Model

Building on Hirschman’s (1970) theory of loyalty, which emphasizes the role of loyalty in shaping employees’ decisions to either exit or engage in voice behavior when confronted with organizational decline, Packer’s (2008) normative conflict model offers a detailed framework for examining how organizational identification may drive voice behavior. The normative conflict model argues that individuals who strongly identify with their group are more likely than those with weaker identification to engage in dissent when the group exhibits behaviors that threaten its positive identity. In line with the concept of voice behavior, in the normative conflict model, dissent is defined as “nonconformist reactions motivated by a desire to change group norms and initiate improvement within a group” (Packer, 2008, p. 54). Therefore, similar to voice behavior, dissent is described as a discretionary behavior aimed at benefiting the group. Although dissent may appear disloyal when it conflicts with the preferences of others in the group, the normative conflict model argues that it is fundamentally a psychologically loyal behavior because it reflects an individual’s genuine concern for the group’s interest. Furthermore, similar to Hirschman’s (1970) theory of loyalty, Packer’s (2008) normative conflict model contends that strongly identified individuals are likely to hold higher status within their organization, which enhances their confidence in the potential influence of their voice. While Hirschman (1970) emphasized loyalty as a motivator for voice, Packer (2008) further extends this perspective by conceptualizing organizational identification as a specific form of loyalty that encourages dissent when group norms jeopardize the organization’s identity or effectiveness. Hence, we conclude that this conceptual integration serves as an appropriate overarching framework for addressing our research questions.

### 2.2. Organizational Identification and Voice Behavior

Building upon the normative conflict model (Packer, 2008), we suggest that organizational identification will be positively related to voice behavior. However, while organizational identification might seem an obvious choice for examining loyalty, Burris et al. (2008) operationalized loyalty as affective commitment and found no relationship between affective commitment and voice. Yet, it is important to note that affective commitment may not be the form of loyalty to predict voice behavior. There has been extensive discussion regarding the distinctions between affective commitment and organizational identification (Ashforth et al., 2008; Lam & Liu, 2014; Meyer et al., 2006; Ng, 2015; Riketta, 2005). Affective commitment refers to a positive emotional bond with the organization (Allen & Meyer, 1990). It implies that employees remain committed to the organization only as long as their positive feelings persist, and their attachment may diminish if conditions arise that negatively impact their well-being in the workplace (Ashforth et al., 2008). A meta-analysis of affective commitment (Meyer et al., 2002) found that, of the work experience variables posited as antecedents to affective commitment, perceived organizational support had the strongest correlation. This finding aligns with the idea that affective commitment is developed and is sustained through an ongoing social exchange, wherein the organization fulfills the employee’s needs, and the employee reciprocates with commitment (Eisenberger et al., 1990, 2001; Rhoades et al., 2001). However, as noted earlier, voice is a potentially risky behavior. It is a discretionary contribution to the organization that may not be reciprocated and may even be punished. It also may arise from dissatisfaction with problematic aspects of the organization, which are unlikely to foster positive feelings or be perceived as supportive. As such, voice is more likely linked to forms of loyalty, such as organizational identification, that do are not contingent on social exchange.

Whereas affective commitment is primarily linked to positive emotions, organizational identification can compass a broader range of emotions (Ashforth et al., 2008; Dutton & Dukerich, 1991; Herrbach, 2006; Huy, 2011; Mackie et al., 2000; Seger et al., 2009). Individuals who strongly identify with their social group may typically feel pride, but they may also experience negative emotions, such as shame or frustration, when the organization is threatened or falls short of expectations (Conroy et al., 2017). These emotions are often group-based, arising from the individual’s perception of the significance an event holds for their social group (Mackie et al., 2000; Seger et al., 2009). Such emotions can drive individuals to take actions on behalf of the group they belong to (Huy, 2011; Mackie et al., 2000; Packer & Chasteen, 2010).

On the basis that organizational identification is dependent less on a contingent emotional bond than on a merging of the self-concept with the organization, Ashforth et al. (2008) asserted prioritizing collective interests over affective commitment. This is supported by meta-analytic findings that organizational identification has a stronger relationship with extra-role behaviors than affective commitment (Riketta, 2005). Packer (2008) explored this extensively in his elaboration of the normative conflict model, arguing that highly identified group members dissent to drive improvements when they believe that a group norm is harmful to the collective. Such individuals are even willing to undertake actions that come at a personal cost if they believe these actions serve the best interests of the group (Louis et al., 2004). This includes staying with the group rather than leaving, even when leaving might offer greater personal benefits (Van Vugt & Hart, 2004).

The willingness to take risks for the group is particularly important in light of research findings that voice behavior is “inherently intimidating and fear-provoking” (Detert & Treviño, 2010, p. 263). Assessing the risks involved in voice behavior often induces negative emotions such as fear and anxiety (Conroy et al., 2017; Detert & Edmondson, 2005; Milliken et al., 2003). Trepidation may emerge not only when challenging leaders (Detert & Edmondson, 2005; Kish-Gephart et al., 2009) but also when interacting with peers, due to fear of isolation (Bowen & Blackmon, 2003). Thus, organizational identification may lead to voice by increasing individuals’ willingness to confront potential negative consequences, which typically serve as psychological barriers to speaking up.

Only a limited number of studies have provided some support for the relationship between individuals’ identification with their work units and voice. For instance, E. W. Morrison et al. (2011) demonstrated a positive relationship between identification with a work team and voice behavior. In addition, Liu et al. (2010) found that organizational identification was a predictor of speaking out to coworkers. Unfortunately, these studies did not examine the possible role of social exchange relationships in voice behavior, leaving the importance of organizational identification relative to social exchange processes less clear. Among the social exchanges within organizations, previous studies have found positive relationships between leader–member exchange and voice (Burris et al., 2008; Park & Nawakitphaitoon, 2018; Van Dyne et al., 2008; Wang et al., 2016) and between perceived organizational support and voice (Ng & Feldman, 2012; Tucker et al., 2008). Therefore, in order to better understand how employees’ organizational identification affects voice, it is essential to simultaneously consider identity and exchange processes. In keeping with this, we include leader–member exchange and perceived organizational support as control variables in our investigation of the relationship between organizational identification and voice.

**Hypothesis** **1.** *Organizational identification is positively associated with voice behavior, controlling for leader–member exchange and perceived organizational support.*

### 2.3. Moderation of the Voice–Performance Relationship by Organizational Identification

Given organizations’ keen interests in extracting maximum effort from their employees, one might assume that “above and beyond” behaviors like voice would consistently lead to positive performance evaluations. After all, even when suggestions are not deemed actionable, they at least signal an effort to contribute to the organization’s well-being. However, findings on the voice–performance relationship have been mixed. A meta-analysis by Thomas et al. (2010) reported a correlation of ρ = 0.30. The confidence interval excluded zero, but the credibility interval did not. The results of prior studies emphasize that the impact of voice on performance evaluations is contingent on a range of factors. These include the type of voice content (Burris, 2012), supervisor–employee congruence in perceptions of voice behavior (Burris et al., 2013), the prosocial values and negative affectivity of those voicing concerns (Grant et al., 2009), role perceptions (Van Dyne et al., 2008), and the knowledge of emotion regulation strategies (Grant, 2013).

A less frequently noted aspect of Hirschman’s (1970) theory of loyalty is his argument that loyalty not only increases the likelihood of employees engaging in voice but also enhances the effectiveness of their voice. Highly identified members of the organization may be more likely to have thought through their concerns and proposals, with an eye toward the organization’s needs rather than just their own. This approach may result in higher quality and more well-rounded suggestions. Furthermore, even if their voice does not directly lead to improvements, voice behaviors by highly identified members might still generate positive impressions, ultimately contributing to their enhanced performance appraisals.

Highly identified members are more likely to conform to the norms of their social groups (Postmes & Spears, 1998; Terry & Hogg, 1996). That is, they are more prototypical (i.e., representing the attributes that set organizational members apart from non-members; Tajfel & Turner, 1979). They tend not to be habitual dissidents (Packer, 2008; Postmes & Spears, 1998), making their dissent more salient. Furthermore, highly identified members might exercise voice in ways that align with the organization’s norms, using language and culturally consistent mannerisms (Hogg & Reid, 2006). Also, Packer (2008) suggested that, because of their loyalty, strongly identified organizational members may refrain from speaking up until a problem has grown significant. Thus, by the time high identifiers engage in voice, it might be easier for others to recognize the validity of their concerns and to understand the content of their message. Finally, high identifiers are more likely to engage in prosocial behavior toward the organization in general (Dukerich et al., 2002; Lee et al., 2015; Riketta, 2005), and some of that behavior is likely to involve actions that demonstrate prioritizing organizational interests over personal concerns, which may be visible to others. These factors may strengthen voice recipients’ belief that the voicer is speaking up with concern for the organization’s best interest in mind (Grant et al., 2009; Prapavessis & Carron, 1997; Whiting et al., 2008). For instance, an experimental scenario study by Whiting et al. (2008) found that people who engaged in high levels of voice were more likely to receive better performance appraisals when they also engaged in high levels of helping. All these factors might aid in the adoption of the ideas of a highly identified member of the organization, but they could also benefit performance appraisals by enhancing perceptions of that person’s prosociality, prototypicality, and status.

The normative conflict model (Packer, 2008) suggests that, whereas highly identified members express concerns constructively and with the intent to uphold a positive group identity, people who are weakly identified exercise voice in a way that is more self-interested and potentially destructive. They tend not only to challenge the specific norms pertaining to the area of concern they are addressing but also to consistently and openly disregard the organization’s broader norms. They seek to actively disengage from the organization, creating distance between themselves and the members and activities of the organization. As a result, their proposals may be of limited utility and are likely to be received defensively or dismissively (Abrams et al., 2000). Because their voice stems from a sense of disengagement from the organization (Packer & Miners, 2012), their work may be suffering. Moreover, the voice exercised by weakly identified people might further highlight their non-prototypicality and their lack of alignment with the organization’s interests. Whether performance is based on actual task performance or attributions of an individual’s commitment, appraisals of weakly identified individuals are likely to suffer when they vocalize ideas and criticisms.

**Hypothesis** **2.** *Organizational identification of voicing individuals will moderate the relationship between their voice behavior and performance evaluation by their supervisors such that the voice–performance relationship is positive for highly identified members and negative for weakly identified members.*

As Hypotheses 1 and 2 represent, we propose that organizational identification may play a critical role both in the voice occurrence process (a predictor of voice behavior) and in the voice outcome process (a moderator for the impact of voice on performance evaluation). In Hypothesis 3, we aim to examine these two different roles of organizational identification in voice processes. We apply Preacher et al.’s (2007) model, which illustrates “the case in which the effect of M [mediator] on Y [dependent variable] is moderated by the independent variable X” (p. 195). This combined process model posits that voice behavior is anticipated to positively mediate the relationship between organizational identification and performance evaluation, particularly when one’s organizational identification is strong. In other words, although organizational identification may linearly increase one’s participation in voice, its benefits in terms of receiving positive evaluations of voice behavior may emerge only when the strength of identification is sufficient to demonstrate a commitment to the organization’s collective goals and to communicate that he/she is a prototypical member.

**Hypothesis** **3.** *Organizational identification will moderate the indirect effect from organizational identification, via voice behavior, to performance evaluation.*

## 3. Method

### 3.1. Sample and Procedures

The participants in this study were 455 cadets at a military educational institute in South Korea. The cadets both work and live together to achieve their educational and training goals. They themselves operate the basic functions of the student organization based on the honor system, and their activities are supervised by commanding officers (company supervisors) who closely observe their behaviors and regularly evaluate their performance. Although military organizations, including military academies, are typically characterized by hierarchies and regulations, there is a growing recognition of the importance of voluntary voice behavior, such as suggesting organizational improvement and raising ethical concerns, among military personnel (Hilverda et al., 2018; Kim & Vandenberghe, 2020; Nye et al., 2022). In addition, because cadets live in a self-managing community, voluntary voice behavior is not only necessary for the effective operations of the community but is also officially encouraged. The organizational system allows cadets to formally and informally participate in voice behavior via several ways such as online bulletin boards, supervisor–cadet meetings, and colleague meetings.

With agreement from the institute, researchers individually approached the cadets to ask for their participation. All available second-, third-, and fourth-year cadets (n = 148, 155, and 152, respectively) were invited to participate. Their participation was voluntary. However, due to a lack of adequate time for supervisors to observe first-year cadets’ behavior, they were not involved in this study. Those who consented to take the survey filled out a first questionnaire that included measures of their own organizational identification, control variables (leader–member exchange and perceived organizational support), and demographic variables. About 15 days after the first survey, participants received a second survey in which they were asked to rate the level of voice behavior of a randomly assigned peer in the same group (company) who was also participating in this study. During the second phase, company supervisors were also asked to rate the performance of survey participants who belonged to their company. Because each company is supervised by two officers, performance appraisals for each participant were obtained from both officers. All surveys were conducted by a paper and pencil method.

After deleting cases that had one or more missing values (i.e., listwise deletion), 442 cases were used for the data analyses. This overall sample size is considered appropriate for the SEM analysis of the research model. It meets the general guideline that at least 200 cases are necessary for SEM analyses (Kline, 2012). Moreover, with the model complexity indicated by 84 free parameters in our model, the calculated N:q ratio, which reflects the ratio of sample size to the number of free parameters, was 5.3:1, exceeding the recommended minimum of 5:1 (Bentler & Chou, 1987). Their average age and average organizational tenure were 20.73 years (SD = 1.18) and 1.99 years (SD = 0.81), respectively, and 91.2% of participants were male.

### 3.2. Measures

Responses to all scale measures, except for performance appraisal, were given on a 5-point Likert scale from strongly disagree = 1 to strongly agree = 5.

*Organizational identification*. Organizational identification was measured with F. Mael and Ashforth’s (1992) 6-item self-report scale. Internal consistency reliability was α = 0.86. Sample items were “I am very interested in what others think about this school”, “This school’s successes are my successes”, and “When someone praises this school, it feels like a personal compliment”.

*Voice behavior*. The voice behavior of each participant was assessed by peer ratings. We adopted three items (α = 0.67) from Van Dyne and LePine’s (1998) original six-item employee voice scale. Some of the items are incompatible with the definition of voice behavior since they do not directly measure verbal behaviors (e.g., “This particular colleague keeps well informed about issues where his/her opinion might be useful to the work group”). Thus, similar to other researchers (Detert & Burris, 2007), this study used only those items that focus on improvement-oriented verbal behavior. The included items are “This particular colleague develops and makes recommendations concerning issues that affect the cadet group”, “This particular colleague communicates his/her opinions about issues in cadet life to other cadets even if his/her opinion is different and others disagree with him/her”, and “This particular colleague speaks up in the cadet group with ideas for new methods or changes in procedures”.

*Performance appraisal.* Three items that evaluate the overall performance of the participants were developed. The items were “In consideration of all aspects, how do you evaluate the overall performance of this cadet?” (1 = poor/minimal pass to 5 = extremely excellent), “Compared with the other cadets in the same year, this cadet’s overall performance is” (1 = very low to 5 = very high), and “How much does this cadet’s performance meet the standards of educational achievement?” (1 = much less than the standard to 5 = much exceeds the standard). The performance of each participant was rated by both of the person’s supervisors. The average internal consistency reliability of the two ratings was 0.96. In addition, the inter-rater reliability was ICC(2,1) = 0.51 (Shrout & Fleiss, 1979). Since there were many cases (n = 119) where one of the two supervisors was not available to complete the survey, we averaged the two supervisors’ ratings for each of the three items.

*Control variables.* Perceived organizational support (POS) and leader–member exchange (LMX) were controlled. While they were expected to be associated with voice behavior (Settoon et al., 1996; Wayne et al., 1997), we controlled for them in the analysis of performance appraisals due to their potential effects on job performance (Armeli et al., 1998; Gerstner & Day, 1997). LMX was measured using the 7-item self-report measure (α = 0.81) of Graen and Uhl-Bien (1995). Sample items were “Regardless of the amount of formal authority the company commander has, he/she would bail me out at his/her expense” and “I have enough confidence in my company commander that I would defend and justify his/her decision if he/she were not present to do so”. In addition, perceived organizational support (POS) was measured with 6 items (α = 0.83; Eisenberger et al., 2001). Sample items were “This institute really cares about my well-being” and “This institute strongly considers my goals and values”. Gender (male = 1, female = 0) and organizational tenure (in years) of the participants were also controlled.

## 4. Results

Table 1 presents the means, standard deviations, and correlations of the variables. To test the hypothesized model, we employed the structural equation model (SEM) with maximum likelihood robust (MLR) estimation in Mplus-7. The advantage of MLR estimation is that it takes into account potential non-normality in the data (Muthén & Muthén, 1998–2012).

### 4.1. Confirmatory Factor Analysis

We first examined the measurement model using confirmatory factor analysis before testing the hypothesized structural model (Anderson & Gerbing, 1988). Results showed that the proposed five-factor measurement model that consists of LMX, POS, OI, voice, and performance evaluation fit the data well, *χ*^2^ (265) = 629.62, CFI = 0.92, RMSEA = 0.06 (90% CI = 0.05, 0.06), and SRMR = 0.05. Moreover, every indicator significantly loaded on its assumed construct factor at *p* < 0.001. We compared the proposed five-factor model with two potentially plausible four-factor measurement models to further check the construct validity. First, POS and LMX can be regarded as one factor, as both reflect the social exchange relationships of organizational members. We tested whether the model separating POS and LMX is better than an alternative model that combined the two. The chi-square difference test demonstrated that our five-factor model is significantly better (∆*χ_scaled_*2(1) = 963.23, *p* < 0.001) than the four-factor model that fixed the inter-factor correlation between POS and LMX at 1 (*χ*^2^ (266) = 1280.84, CFI = 0.77, RMSEA = 0.09; 90% CI = 0.09, 0.10, SRMR = 0.09). Second, because both POS and organizational identification may reflect connectedness between individuals and the organization, one might argue that POS and organizational identification can be treated as one factor. The chi-square difference test, however, revealed that the current model discriminating POS and organizational identification fit the data significantly better (∆*χ_scaled_*2(1) = 376.05, *p* < 0.001) than the reduced four-factor model (*χ*^2^ (266) = 1256.73, CFI = 0.78, RMSEA = 0.09, 90% CI = 0.09, 0.10, SRMR = 0.08).

In addition to the comprehensive assessment, we used specific indicators, such as composite reliability (CR) and average variance extracted (AVE), to evaluate the construct validity. CR, another indicator of internal consistency reliability, is particularly suitable for use in SEM (Cheung et al., 2024). Although CR measures reliability, good reliability (above 0.7) is also regarded as a requirement of convergent validity (Cheung et al., 2024; Fornell & Larcker, 1981). AVE, which represents the proportion of variance in the observed variables explained by the latent construct, is an indicator of convergent validity (Hair et al., 2017). While an AVE value above 0.5 is generally considered acceptable, it is important to note that this is an arbitrary threshold and not an absolute rule (Cheung et al., 2024). As shown in Table 1, the AVE values of some variables were slightly below 0.5. However, the CR values of most constructs exceeded 0.7, except for voice, which was measured by only three items. Moreover, discriminant validity was evaluated by comparing the square root value of AVE (SQAVE) of each construct with its latent correlations with other constructs in the model. Fornell and Larcker (1981) suggest that the SQAVE of a construct should exceed its correlation with any other construct. Table 1 shows that all constructs satisfied this requirement.

In sum, although the AVE values for some variables fell below the recommended threshold, the comprehensive assessment of the measurement model, including comparisons with alternative models and evaluations of other validity indices, provides compelling evidence for the overall validity of the measurement model.

### 4.2. Hypothesis Testing

**Figure 1:**
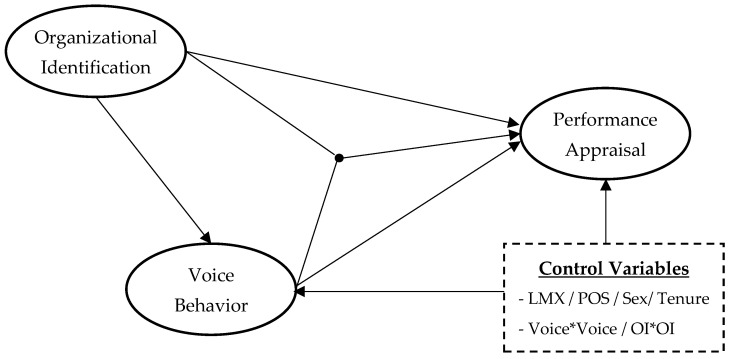
Structural equation model.

In order to test the hypotheses, we followed the three steps suggested by Hayes and his colleagues (Hayes, 2013; Preacher et al., 2007). Although their analytic approaches are mostly based on regressions, we adopted SEM analyses to account for measurement error. Figure 1 illustrates the structural portion of the SEM model.

Step 1.
$Voice\;Behavior=a_{0}+a_{1}OI+\sum a_{2i}{covariate}_{i}+e_{voice}$

Step 2.
$Performance\;Appraisal=b_{0}+c^{\prime }OI+b_{1}Voice+b_{2}OI\cdot Voice+b_{3}OI\cdot OI+b_{4}Voice\cdot Voice+\sum b_{5i}{covariate}_{i}+e_{Performance}=b_{0}+c^{\prime }OI+\left(b_{1}+b_{2}OI\right)Voice+b_{3}OI\cdot OI+b_{4}Voice\cdot Voice+\sum b_{5i}{covariate}_{i}+e_{Performance}$

Step 3. Conditional Indirect Effect: $a_{1}\left(b_{1}+b_{2}OI\right)$

First, the effect of organizational identification on voice behavior was tested with the covariates—LMX, POS, gender, and organizational tenure—controlled. Second, the moderating effect of organizational identification on the relationship between voice behavior and performance appraisal was examined, controlling for the direct effect of organizational identification on performance appraisal (*c*’). The interaction effect of the two latent constructs, voice and organizational identification, was estimated by Klein and Moosbrugger’s (2000) latent moderated structural equation (LMS) method. This method is based on maximum likelihood estimation and models the non-linear effect (e.g., interaction effect and quadratic effect) of latent variables by directly creating their latent product term without product indicators of the latent factor. The LMS method is advantageous in that it considers non-normality caused by latent interaction effects, producing relatively unbiased estimates (Klein & Moosbrugger, 2000; Marsh et al., 2013). In this latent moderation effect analysis, quadratic latent terms of voice behavior and organizational identification, along with the covariates, were also included as control variables due to the potential confounding effects of the curvilinearity of the variables in interaction (Edwards, 2009). Finally, the previous two procedures allowed us to calculate the conditional indirect effect: a_1_ (b_1_ + b_2_ *OI*). It basically integrates the voice occurrence process (the antecedent of voice behavior) and the conditional voice outcome process and investigates whether voice behavior mediates organizational identification and performance appraisal in different manners depending on the extent of organizational identification. The changing nature (e.g., direction, significance, and confidence interval) of the mediation effects was examined by applying low (one standard deviation below the mean), medium (mean), and high (one standard deviation above the mean) values of organizational identification to the indirect effect term (Preacher et al., 2007).

**Figure 2:**
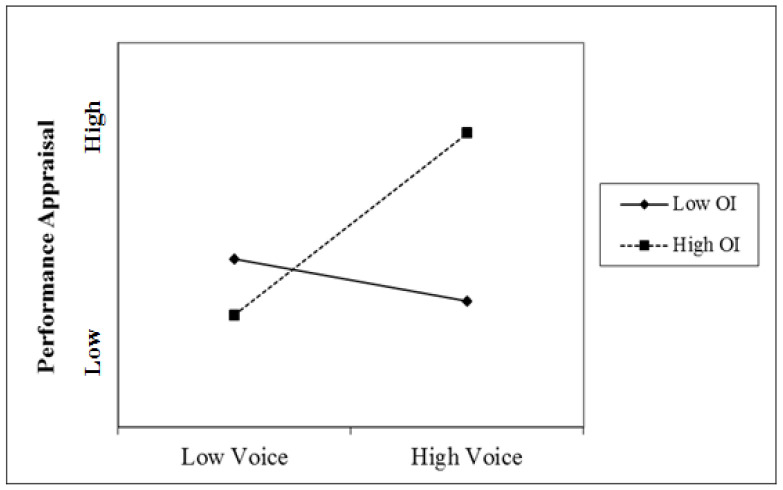
The moderating effect of organizational identification on the relationship between voice and performance appraisal.

Table 2 describes the results of the analysis. As Hypothesis 1 proposed, organizational identification incrementally predicted voice behavior above and beyond the two social exchange variables, LMX and POS. After controlling for the covariates, organizational identification had a significant positive relationship with voice behavior (*β* = 0.28, *p* < 0.001), thus supporting the hypothesis. In addition, organizational identification was found to have a strong moderating effect on the relationship between voice and performance appraisal (*β* = 0.16, *p* < 0.01). To help interpret the moderation effect, the association between voice and performance appraisal was plotted for high (one standard deviation above the mean) and low (one standard deviation below the mean) values of organizational identification (Figure 2). Simple slopes analyses were also performed for these two plotted regression lines (Aiken & West, 1991). For those high in organizational identification, voice and performance appraisal were positively associated (*β* = 0.26, *p* < 0.01). However, for those low in organizational identification, voice behavior was not significantly related to performance appraisal (*β* = −0.06, *p* = 0.50), and the slope even showed a negative tendency, supporting Hypothesis 2.

Finally, Hypothesis 3 proposed that the indirect effect of organizational identification on performance appraisal, via voice behavior, would differ by the strength of organizational identification. From the first two steps, the following indirect effect term could be obtained: 0.28 (0.10 + 0.16 * *OI*). The conditional indirect effect was investigated using three different values of organizational identification. As Table 3 shows, the indirect effect estimation by the first-order delta method (Sobel, 1982, 1987) indicated that, when the strength of organizational identification was at low and medium levels, the mediation effect of voice behavior was not significant and their 95% confidence intervals contained zero. On the other hand, when organizational identification was at a high level, it was significantly positive and the 95% confidence interval did not contain zero.

Although the delta method is widely used for indirect effect estimation, it might present inappropriate confidence intervals due to its unrealistic normality assumption about the distribution of the point estimate of the indirect effect (MacKinnon et al., 2002). For this reason, we also constructed confidence intervals using Monte Carlo analyses, which do not assume the normality of the indirect effect estimate (Hayes & Preacher, 2013; Preacher & Selig, 2012). Monte Carlo analyses repeatedly generate estimates of the component parameters (e.g., a and b) of the indirect effect (e.g., ab), assuming that the point estimates of the components are normally distributed, and produce confidence intervals based on the actual distribution of the product term (ab) calculated by the generated component estimates (Preacher & Selig, 2012). We computed the Monte Carlo confidence intervals from 100,000 simulated generations of the component estimates and found that they were almost identical to the confidence intervals we found above (see Table 3). The 95% confidence intervals of the indirect effect were positive and did not include zero under high organizational identification but included zero for those with low and medium identifications. Consequently, the changing pattern of the indirect effect revealed the existence of meaningful conditional indirect effects, providing support for the moderating role of organizational identification in the organizational identification–voice behavior-performance appraisal process (i.e., Hypothesis 3). Hence, the result indicates that voice behavior mediates organizational identification and performance evaluation only when identification is strong enough to expose the person’s oneness with the organization.

## 5. Discussion

### 5.1. General Discussion

In this research, organizational identification not only enhanced organizational members’ engagement in voice behavior but also contributed to a positive relationship between their voice behavior and manager performance evaluations. On the other hand, in addition to engaging in less voice behavior, low identifiers received no benefits from their voice. The results support Hirschman’s (1970) contention that loyal organizational members are more likely to be effective voicers.

In contrast, social exchange appeared to have no effect on voice in our model; neither perceived organizational support nor leader–member exchange showed a statistically significant relationship with voice. This contradicts previous theorizing and empirical findings. Extra-role behaviors are often explained through a social exchange lens (Organ, 1988), and the prediction that leader–member exchange would influence voice is based on the idea that employees wish to reciprocate support and respect from their supervisors by going above the call of duty (Van Dyne et al., 2008). Prior research examining this relationship has not considered social identity processes. However, our findings suggest that it is the sense of shared values and a common fate with one’s organization, perhaps rooted in a positive supervisory relationship but not specifically because of that relationship, that catalyzes voice behaviors.

The findings regarding the effect of organizational identification on the relationship between voice and performance evaluations from managers suggest that managers evaluate not only the content of the voicing message but also the source and, perhaps, the style of delivery. This is consistent with findings that employees engaging in proactive behaviors, such as voice behavior, receive better performance evaluations when they are generally low in negative affect at work, engage in higher levels of helping, and are knowledgeable about emotion regulation strategies (Grant, 2013; Grant et al., 2009). Interestingly, a supplemental analysis of our data revealed that organizational identification did not have a significant indirect effect on performance evaluation through voice without consideration of the moderating role of organizational identification itself (standardized estimate of indirect effect = 0.02, *p* = 0.24). In light of this, it seems that high identifiers’ tendencies to engage in more voice are not sufficient for them to receive more favorable performance evaluations. Rather, it is more likely their prototypicality and the way they communicate their concerns or ideas that draw the approbation of their managers.

### 5.2. Theoretical and Practical Implications

Our findings indicate that identity-based theories like the normative conflict model (Packer, 2008) can offer fruitful ways to understand the processes surrounding voice. It seems likely that social identity permeates the voice process. We have already demonstrated that organizational identification can influence the level of engagement in voice and the relationship between voice and performance evaluation. The normative conflict model (Packer, 2008) also suggests that organizational identification could influence the types of issues that people identify as worth speaking up about, how soon they bring the issue up, their motives for voice, and whether the voice is expressed privately or publicly. The drawback of applying the normative conflict model to voice is that the model rather narrowly focuses on the expression of nonconforming minority opinions. It appears as if the research literature implicitly frames voice in this way, although there has been growing attention to distinctions among different types of voice (Burris, 2012; Liang et al., 2012; Maynes & Podsakoff, 2014). This stream of research has yet to address the degree to which voice is conformist or reflective of a minority view, but it seems likely that these factors would be influential. Bowen and Blackmon (2003) theorized that, due to the threat of isolation, people will be less likely to exercise their voice when their opinion is in the minority and when they are not members of the ingroup. Although Bowen and Blackmon were specifically referring to people who were demographic or stigmatized minorities, the argument may apply more broadly to people who are non-prototypical or who hold a low identification with their group.

Of course, isolation is also a threat to highly identified group members, according to Packer (2008), since they derive a great degree of their sense of self and belonging from the group. That is why the normative conflict model proposes that high identifiers will maintain an uneasy silence when confronted with situations that call for voice until the discomfort becomes overwhelming. Yet, there are research findings showing that holding a minority viewpoint enhances self-concept clarity among people who are strongly identified with the group (K. R. Morrison & Wheeler, 2010). Thus, high identifiers might derive sufficient personal fulfillment from voicing minority positions, making them more likely to do so than low identifiers. It could also be the case, however, that high identifiers are more likely to exercise voice when they know that their position is shared by a majority of members, communicating widely held but little-stated concerns or ideas.

There is already evidence that organizational identification influences the reaction to information, indicating that change is needed in the organization (Dutton & Dukerich, 1991). In their case study of the Port Authority’s response to the problem of the presence of homeless people in the transit system, Dutton and Dukerich (1991) found that the organization acted on issues that harmed its image and that the actions taken were also bound by the organization’s identity. This points to a role for social identity processes on both sides of the voice equation. There is still very little research on how managers react to voice, particularly on the role of managers’ self-concept. One recent paper found that managers with low managerial self-efficacy were more resistant to receiving voice because they did not feel they had the competencies to be responsive (Fast et al., 2013). A study of the development of professional identity among medical residents (Pratt et al., 2006) suggests that identity informs people’s sense of competence at work, a notion codified in Ashforth et al.’s (2008) model of identification. Thus, organizational identification could be as important for whether managers solicit, welcome, and act on voice as it is for whether employees offer and benefit from voice.

From a practical standpoint, because so little is known about how organizational identification influences voice and voice outcomes, it seems premature to urge managers to try to increase organizational identification if they want to receive more voice. Of course, organizational identification is already linked to several positive outcomes for organizations, including discretionary behaviors (Lee et al., 2015; Riketta, 2005), and may be worth promoting for that reason. However, it is important to better understand the mechanisms underlying the relationships among organizational identification, voice, and performance evaluations. If anything, managers should be aware that the people exercising their voice most often could be those who are most identified with the organization. It is not yet clear that the higher performance evaluations resulting from voice by high identifiers are a result of their having better ideas. People who are not strongly identified with the organization may offer valuable and unique insights. Moreover, as Bowen and Blackmon (2003) pointed out, members of demographic minorities and stigmatized groups may be more likely to remain silent because their social identity conflicts with the organization’s identity. If organizations want to ensure that a variety of perspectives are included in their decision making, it might be necessary to make concerted efforts to ensure that people who are non-prototypical are heard. In an organization that is trying to change, these members may be just the types of employees the organization needs and wants to retain. They may have the skills, norms, and values that the organization is attempting to shift toward, rather than being wedded to the current culture. Furthermore, giving low identifiers space to voice their concerns, addressing those concerns in some way, and ensuring that they are rewarded for contributing might increase their identification.

Our research also provides practical implications for employees. People considering engaging in voice could consider trying to communicate their loyalty to the organization when voicing. Rather than framing their concerns in a self-interested manner, it could be more effective to appeal to the core values and defining characteristics of the organization. Prior evidence suggests that communicating voice in an emotionally positive manner yields better performance evaluations (Grant et al., 2009), possibly because people who express emotions like pride and enthusiasm are perceived as more identified with the organization. When expressing strong identification seems unrealistic—for instance, for individuals whose reputation for nonconformity would make a display of loyalty seem difficult to believe—people with concerns or ideas might try to find allies among those who are known as loyal members and encourage them to speak up.

### 5.3. Strengths, Limitations, and Future Research

This study has numerous strengths. We collected data from multiple sources and measured organizational identification and the control variables at a separate time from voice and performance. To account for social exchange processes, we controlled for variables at the level of the relationship between the organization and the individual (i.e., perceived organizational support) and between the leader and the individual (i.e., leader–member exchange). We also adopted SEM analyses that accounted for measurement error (Hayes & Preacher, 2013). Finally, we tested our model in a military organization setting. Because military organizations tend to foster high levels of organizational identification (F. A. Mael & Ashforth, 1995), this likely provided a conservative test of our hypotheses, as we might expect greater variation in levels of organizational identification in settings with less distinctive cultures (Ashforth et al., 2008).

As is typical, this study also has several limitations. First, the military organization setting may have limited the generalizability of our findings. It is based on an overwhelmingly male military educational setting in Korea and, therefore, is different from the types of work settings in the United States in which most published research on voice behaviors has been conducted. However, we believe that our sample provides a relevant setting for testing our research model, as the cadets have ample formal and informal opportunities to engage in voice behavior and are also subject to evaluations of their performance. Nevertheless, future research is needed to examine whether these findings apply to different organizational and cultural contexts.

Second, we proposed, but did not test, several processes that may underlie the relationships among voice, organizational identification, and supervisor performance evaluations. As discussed earlier, it is important to gain insight into these processes to better understand the reasons behind the effects observed here. In particular, experimental research is necessary to clarify causal relationships.

Third, in this study, voice behavior was rated by peers. Peer ratings may not accurately reflect managers’ perceptions of an individual’s voice. Nevertheless, using peer measurements avoided potential same-source bias in the relationship between voice and performance evaluations. Moreover, we did not measure different types of voice. Burris (2012) found that a voice that challenges the status quo (i.e., challenging voice) is negatively associated with performance evaluations because it diminishes perceptions of loyalty and increases threat perceptions. Meanwhile, supportive voice, measured by Burris using the items from the Van Dyne and LePine (1998) scale that were omitted from this study, was positively associated with performance evaluations, loyalty perceptions, and even lower threat perceptions. Thus, Burris concluded that the type of voice can affect performance evaluations. However, supportive voice is inconsistent with the definition of voice as verbal behavior that challenges the status quo (Detert & Burris, 2007; Van Dyne & LePine, 1998). Supportive voice might represent a different construct altogether. Voice behaviors from Van Dyne and LePine’s (1998) voice scale, used by Burris to operationalize supportive voice, such as “I get involved in issues that affect the quality of work-life here”, could be construed as in-role or helping behaviors. However, regardless of how supportive voice aligns with the definition of voice behavior, the items that we used were those considered by Burris to describe challenging voice behaviors. Thus, within the framework Burris advanced, our findings provide solid substantiation for the argument that organizational identification overcomes many of the perils of speaking up.

Fourth, this study focuses primarily on prosocial motives as the key driver of voice behavior, which may overlook the complexity of the various motivations that exist in organizational contexts. While the alignment between prosocial motives and organizational identification was central to this study, self-interest motives—such as those related to career advancement or reputation building—were not explicitly examined, despite the fact that they may coexist with and influence prosocial motives (Choi et al., 2015; Fuller et al., 2007). Additionally, the alignment of collective interests and self-interests is often the result of a prolonged learning process in which individuals come to realize that cooperating with collective interests can ultimately serve their personal interests (Van Knippenberg & Sleebos, 2006). This idea is consistent with the social identity theory, suggesting that, as individuals identify more strongly with the organization, they may perceive the organization’s success as intrinsically tied to their own success, thereby aligning their self-interest with collective goals (Ashforth & Mael, 1989). Previous studies also provide support in that individuals are more likely to engage in behaviors that benefit the organization, such as voice, when they see these behaviors as being consistent with their personal values and goals (E. W. Morrison, 2014). Thus, as individuals learn to align their personal goals with those of the organization, their motivation to support the organization or engage in voice behavior may increase, facilitating a more seamless integration of self-interest and collective interest. Future research could benefit from simultaneously examining both prosocial and self-interest motives, providing a more comprehensive understanding of how these motives influence voice behavior and managerial evaluations of performance. Incorporating both types of motives would offer a more balanced and realistic view of the dynamics of voice behavior within organizations.

Finally, in this study, we could not directly assess the fit statistics of our structural equation model because fit statistics are not provided by the latent moderated structural equation modeling method (Kline, 2012). However, the unconditional indirect model, which does not include the moderation effect of organizational identification, fits the data well (*χ*^2^ (311) = 750.71, *p* < 0.01, CFI = 0.91, RMSEA = 0.06 (90% confidence interval: 0.05, 0.06), SRMR = 0.05). Based on this, we infer that the current conditional mediation model, which more appropriately explains relationships among study variables than the unconditional mediation model, would also fit the data well.

## 6. Conclusions

The findings of this study suggest that loyalty operates in the voice process as Hirschman (1970) proposed. It not only prompts voice but also makes one’s voice more palatable to recipients. We demonstrated that challenging existing practices while expressing one’s loyalty is effective not only for politicians but also for members of organizations. As noted earlier, prior research has demonstrated that people often withhold their voice due to perceived risks (Detert & Edmondson, 2011). However, organizations need their employees to take these risks. This research underscores the potential value of fostering strong identification with the organization to encourage such risk taking, with the caveat that organizations need also to consider that biases toward highly identified organizational members may exclude valuable input from those on the margins.

## Figures and Tables

**Table 1:** Means, standard deviations, and correlations.

	Mean	SD	1	2	3	4	5	6	7	CR	AVE
1. Sex	0.91	0.28	-								
2. Organizational tenure	1.99	0.81	−0.01	-							
3. Leader–member exchange	3.63	0.62	0.04	0.02	(0.66)	0.29 ***	0.23 ***	−0.02	0.07	0.82	0.44
4. Perceived organizational support	3.13	0.76	−0.04	−0.12 *	0.29 ***	(0.73)	0.41 ***	0.12	0.11 *	0.85	0.53
5. Organizational identification	4.23	0.69	0.06	−0.08	0.17 ***	0.35 ***	(0.71)	0.27 ***	0.10 *	0.86	0.51
6. Voice	3.01	0.84	0.02	−0.01	−0.03	0.09	0.19 ***	(0.65)	0.10	0.68	0.42
7. Performance appraisal	3.25	0.98	−0.03	−0.02	0.07	0.10 *	0.08	0.09	(0.94)	0.96	0.88

Note: The values above the diagonal represent correlations between latent constructs; * *p* < 0.05, *** *p* < 0.001. Square root values of average variance extracted (SQAVE) are within the parentheses on the diagonal.

**Table 2:** Results of step 1 and step 2 analyses of the structural model.

Mediator/Dependent Variable	Predictor	Standardized Estimate (*β*)	Standard Error (S.E.)	*β*/S.E.	95% CI	
					Lower	Upper
Step 1. Mediator: Voice						
	Sex	0.01	0.21	0.07	−0.41	0.43
	Organizational Tenure	0.00	0.07	−0.01	−0.14	0.14
	LMX	−0.10	0.07	−1.48	−0.23	0.03
	POS	0.03	0.07	0.46	−0.11	0.18
	OI (a_1_)	0.28 ***	0.08	3.49	0.12	0.44
Step 2. Dependent Variable: Performance Appraisal						
	Sex	−0.13	0.18	−0.74	−0.49	0.22
	Organizational Tenure	−0.02	0.06	−0.30	−0.14	0.10
	LMX	0.04	0.06	0.81	−0.06	0.15
	POS	0.06	0.06	0.94	−0.06	0.18
	OI	0.08	0.08	0.91	−0.09	0.25
	Voice (b_1_)	0.10	0.07	1.45	−0.04	0.23
	OI*OI	−0.01	0.04	−0.34	−0.08	0.06
	Voice*Voice	−0.09 *	0.04	−2.16	−0.18	−0.01
	OI*Voice (b_2_)	0.16 **	0.05	2.98	0.05	0.27

Note: * *p* < 0.05, ** *p* < 0.01, *** *p* < 0.001; OI*OI: latent quadratic term of organizational identification; Voice*Voice: latent quadratic term of Voice.

**Table 3:** Conditional indirect effects.

Level of Organizational Identification	Standardized Estimate of Indirect Effect (*β*)	Standard Error (S.E.)	*β*/S.E.	*p*-Value	95% Confidence Interval	
					Lower	Upper
Low (mean − 1 SD)	−0.02	0.02	−0.67	0.50	−0.06 (−0.07)	0.03 (0.03)
Medium (mean)	0.03	0.02	1.30	0.19	−0.01 (−0.01)	0.07 (0.08)
High (mean + 1 SD)	0.07	0.03	2.11	0.03	0.01 (0.02)	0.14 (0.15)

Note: the values in parentheses below confidence intervals represent Monte Carlo confidence intervals.

## Data Availability

The data presented in this study are available on request from the first author.
